# Synthesis of a highly water-soluble acacetin prodrug for treating experimental atrial fibrillation in beagle dogs

**DOI:** 10.1038/srep25743

**Published:** 2016-05-10

**Authors:** Hui Liu, Ya-Jing Wang, Lei Yang, Mei Zhou, Man-Wen Jin, Guo-Sheng Xiao, Yan Wang, Hai-Ying Sun, Gui-Rong Li

**Affiliations:** 1 Department of Medicine, Li Ka Shing Faculty of Medicine, University of Hong Kong, Pokfulam, Hong Kong, China; 2 Department of Pharmacology, School of Basic Medicine, Tongji Medical College, Huazhong University of Science and Technology, Wuhan, 430030 China; 3 Department of Pharmacy Engineering Specialty, College of Chemistry and Chemical Engineering, Guangxi University, Nanning, Guangxi, 530004 China; 4 Department of Anesthesiology, Union Hospital, Tongji Medical College, Huazhong University of Science and Technology, Wuhan, 430022 China; 5 Xiamen Cardiovascular Hospital, Medical College of Xiamen University, Xiamen, Fujian, 361004 China

## Abstract

We previously reported that duodenal administration of the natural flavone acacetin can effectively prevent the induction of experimental atrial fibrillation (AF) in canines; however, it may not be used intravenously to terminate AF due to its poor water-solubility. The present study was to design a water-soluble prodrug of acacetin and investigate its anti-AF effect in beagle dogs. Acacetin prodrug was synthesized by a three-step procedure. Aqueous solubility, bioconversion and anti-AF efficacy of acacetin prodrug were determined with different methodologies. Our results demonstrated that the synthesized phosphate sodium salt of acacetin prodrug had a remarkable increase of aqueous solubility in H_2_O and clinically acceptable solution (5% glucose or 0.9% NaCl). The acacetin prodrug was effectively converted into acacetin in *ex vivo* rat plasma and liver microsome, and *in vivo* beagle dogs. Intravenous infusion of acacetin prodrug (3, 6 and 12 mg/kg) terminated experimental AF without increasing ECG QTc interval in beagle dogs. The intravenous LD_50_ of acacetin prodrug was 721 mg/kg in mice. Our preclinical study indicates that the synthesized acacetin prodrug is highly water-soluble and safe; it effectively terminates experimental AF in beagle dogs and therefore may be a promising drug candidate for clinical trial to treat patients with acute AF.

Atrial fibrillation (AF) is the most common sustained cardiac arrhythmia and contributes to the increase of morbidity and mortality in the aging population around the world^1^. Although catheter ablation is an effective rhythm-control strategy, drug therapy is still necessary as routine treatment to terminate acute AF or prevent AF recurrence in clinic^2^. However, the proarrhythmia, non-cardiac toxicities and non-atrial selectivity of currently available anti-arrhythmic drugs are a problem for the long-term administration^3^,^4^. Therefore, considerable research efforts are required to discover and develop new safe and effective antiarrhythmic drugs with atrial selective multiple ion channel blockade to manage patients with AF^5^,^6^,^7^.

Several atrial-selective ion channel currents including I_Kur_ (ultra-rapidly delayed rectifier potassium current, encoded by Kv1.5), I_KACh_ (acetylcholine-activated K^+^ current) and also small conductance calcium-activated potassium current (SK_Ca_, encoded by SK1/2/3) are predominantly expressed in atria and are considered to be atrial-selective targets for developing atrial selective anti-AF drugs;^2^,^8^,^9^ however, there is no such drug available in the market. In previous studies, we demonstrated that acacetin, a natural flavone initially isolated from the Chinese medicinal herb Tianshanxuelian (*Snow lotus*), can uniquely inhibit atrial potassium currents including atrial I_Kur_ and I_KACh_, as well as cardiac I_to_ (transient outward potassium current)^10^,^11^,^12^. In a recent study, we found that acacetin could also block SK_Ca_ channels expressed in HEK 293 cells^13^. As expected, acacetin was demonstrated to prevent the induction of experimental AF in anesthetized canines after duodenal administration^10^,^14^. These studies suggest that acacetin may act as a highly potential drug candidate for treating AF by specifically blocking multiple atrial ion channels. However, the poor water solubility of acacetin is a key challenge in developing clinically usable forms.

The prodrug strategy is commonly employed to overcome poor water solubility of hydrophobic drugs in the early stage of drug discovery, especially those that cannot perform very well by formulation strategies. To improve the water solubility of parent drug in the prodrug design, the conjugation of some polar functional groups, e.g. carboxylic, hydroxyl, amine, phosphate, carbonyl, or thiol groups directly or through a linker motif, is a popular choice^15^,^16^,^17^. The present study synthesized a water-soluble prodrug of acacetin by introducing a phosphate group for intravenous use and investigated whether the prodrug could be converted into the parent compound acacetin and terminate experimental AF in beagle dogs. Our results demonstrated that the phosphate sodium prodrug of acacetin was highly water-soluble, could be converted into acacetin *in vivo*, and can effectively terminate AF induced by vagal nerve stimulation in beagle dogs.

## Results

### Synthesis of acacetin prodrug

**Figure 1:**
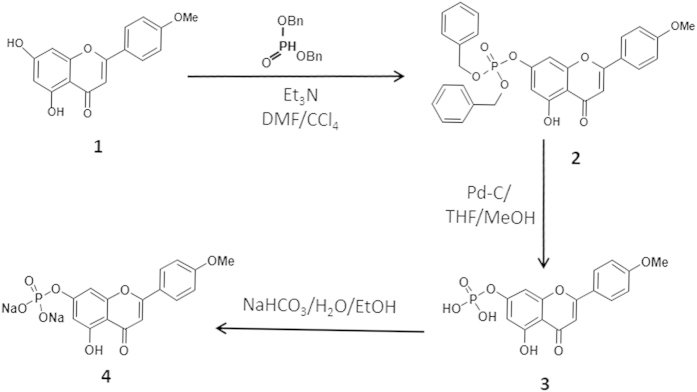
Synthesis scheme of acacetin prodrug. Compound 1, acacetin. **Compound 2**, intermediate phosphate derivative of acacetin. **Compound 3**, phosphate ester of acacetin. **Compound 4**, phosphate sodium salt of acacetin (acacetin prodrug).

Acacetin prodrug was synthesized through three steps as shown in Fig. 1. The chemical structures of intermediate compound 2 and 3 were confirmed by NMR separately. Compound 2 is a yellow solid. Yield 54%. C_30_H_25_O_8_P (544.49). EI-MS: m/z 545.14 [M^+^]. ^1^H NMR (400 MHz, CDCl_3_): δ = 12.81 (s, 1 H), 7.83 (d, J = 8.8 Hz, 2 H), 7.03 (d, J = 8.8 Hz, 2 H), 6.84 (s, 1 H), 6.62 (s, 1 H), 6.54 (s, 1 H), 5.19 (s, 2 H), 5.17 (s, 2 H), 3.91 (s, 3 H) ppm. The NMR spectra of compound 2 is shown in Fig. S1.

Compound 3 is a yellow solid. Yield 92%. C_16_H_13_O_8_P (364.24). EI-MS: m/z 365.04 [M^+^], 285.07 [M + H-P(O)(OH)_2_]^+^. ^1^H NMR (400 MHz, *d*_6_-DMSO): δ = 12.91 (bs, 1 H), 8.06 (d, J = 8.8 Hz, 2 H), 7.11 (d, J = 8.8 Hz, 2 H), 7.01 (s, 1 H), 6.96 (s, 1 H), 6.62 (s, 1 H), 3.87 (s, 3 H) ppm. ^13^C NMR (100 MHz, CDCl_3_): δ 182.04, 163.74, 162.47, 161.15, 159.58, 156.54, 128.37, 122.74, 114.63, 106.05, 103.83, 102.64, 97.64, 55.55 ppm. The NMR spectra of compound 3 is shown in Fig. S2. We finally obtained compound 4 as a light yellow powder with a 92% yield. The chemical structure was confirmed by MS and ^1^H NMR (shown in Fig. S3). The purity (>99%) was determined by HPLC as shown in Supplemental Materials (Fig. S4).

### Aqueous solubility of acacetin prodrug

**Figure 2:**
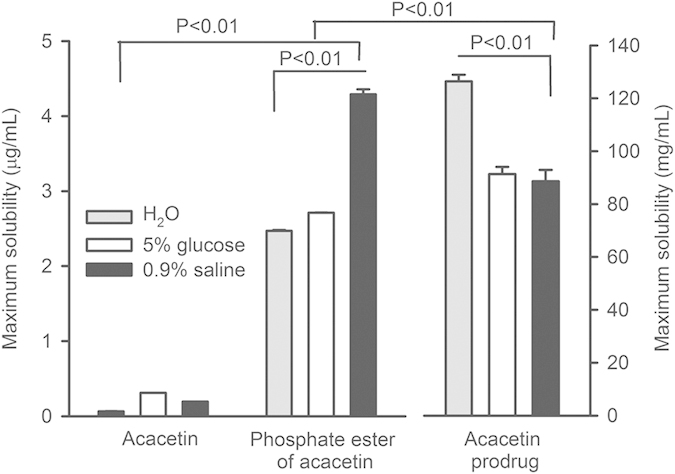
Solubility of acacetin, phosphate ester of acacetin and acacetin prodrug in H_2_O, 5% glucose and 0.9% NaCl, respectively.

The solubility of acacetin (compound 1), phosphate ester of acacetin (compound 3), and acacetin prodrug (compound 4) was determined in H_2_O and the clinically acceptable solutions of 5% glucose and 0.9% NaCl. Figure 2 shows the mean values of solubility of acacetin, phosphate ester of acacetin and acacetin prodrug in different vehicles. The maximum solubility was 64.4 ± 10.9, 312.0 ± 12.1 and 195.0 ± 13.5 ng/mL for acacetin; 2.5 ± 0.1, 2.7 ± 0.1 and 4.3 ± 0.1 μg/mL for phosphate ester of acacetin (n = 3, P < 0.01 vs. acacetin); while 126.4 ± 2.5, 91.4 ± 2.7 and 88.6 ± 4.3 mg/mL for acacetin prodrug (n = 3, P < 0.01 vs. acacetin or phosphate ester of acacetin) respectively in H_2_O, 5% glucose and 0.9% NaCl. Although the aqueous solubility of phosphate ester of acacetin was increased by 37.8, 7.6 and 21 folds respectively in H_2_O, 5% glucose and 0.9% NaCl, it is not sufficient to satisfy the requirement for intravenous administration. Compared to the parent compound acacetin, the solubility of acacetin prodrug was more than 1.9 million folds higher in H_2_O, and more than forty five thousand folds higher in the clinically acceptable solution 5% glucose or 0.9% NaCl. The high water-solubility of the acacetin prodrug would be suitable for developing intravenous preparation and further studies.

### Effects of acacetin prodrug on several cardiac potassium channels

**Figure 3:**
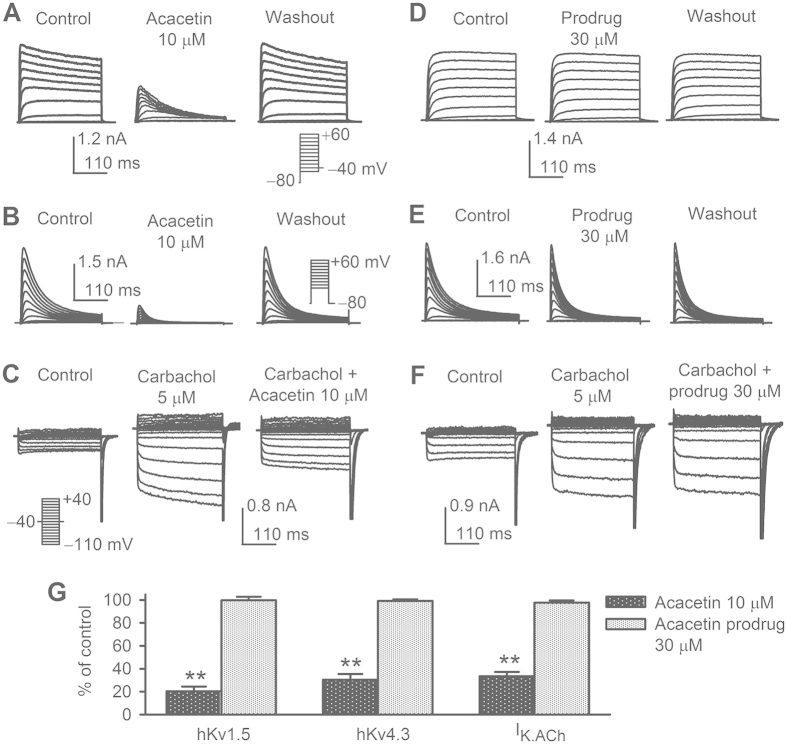
Effects of acacetin and acacetin prodrug on hKv1.5 current, hKv4.3 current, and I_KACh_. (**A**) Acacetin (10 μM) reversibly blocked hKv1.5 current in a representative HEK 293 cell expressing *KCN5A*. (**B**) Acacetin (10 μM) reversibly inhibited hKv4.3 current in a representative HEK 293 cell expressing *KCND3*. (**C**) I_KACh_ elicited by carbachol (5 μM) was decreased by Acacetin (10 μM) in a representative rat atrial myocyte. (**D**) Acacetin prodrug (30 μM) didn’t block hKv1.5 current in a representative HEK 293 cell expressing *KCN5A*. (**E**) Acacetin prodrug (30 μM) had no inhibitory effect on hKv4.3 current in a representative HEK 293 cell expressing *KCND3*. (**F**) Acacetin prodrug (30 μM) had no effect on I_KACh_ elicited by carbachol (5 μM) in a representative rat atrial myocyte. Similar results were obtained in other 4 cells for each group. **(G)** Mean percentage values of 10 μM acacetin and 30 μM acacetin prodrug for inhibiting hKv1.5 current (at + 50 mV), hKv4.3 current (at + 50 mV) and I_K.ACh_ (at −100 mV). n = 5 experiments for each group, **P < 0.01 vs. control.

To determine whether acacetin prodrug, as the parent compound acacetin, would exert inhibitory effects on atrial-specific potassium currents (I_Kur_ and I_KACh_) as well as cardiac I_to_^10^,^11^,^12^, whole-cell current was recorded in HEK 293 cells expressing hKv1.5 gene (encoding for I_Kur_) or hKv4.3 gene (encoding for I_to_), and in rat atrial myocytes. Figure 3 illustrates the voltage-dependent current traces of hKv1.5 and hKv4.3 in HEK 293 cells, and rat atrial I_KACh_ elicited by 5 μ M carbachol in the absence and the presence of 10 μM acacetin (Fig. 3A–C) or 30 μM acacetin prodrug (Fig. 3D–F). As reports described previously^10^,^11^,^12^, acacetin remarkably inhibited hKv1.5 current (Fig. 3A), hKv4.3 current (Fig. 3B) and I_KACh_ (Fig. 3C); however, acacetin prodrug (Fig. 3D–F) had no such effect. Figure 3G illustrates the percentage values of acacetin and acacetin prodrug for inhibiting hKv1.5 current (at + 50 mV), hKv4.3 current (at + 50 mV) and rat atrial I_KACh_ (at −100 mV). Acacetin (10 μM) decreased hKv1.5 current (measured from 0 to the current at end of the voltage step) by 79.7 ± 4.1% (n = 5, P < 0.01 vs. control), hKv4.3 current (measured from 0 to the current peak) by 69.3 ± 5.1% (n = 5, P < 0.01 vs. control), and I_K.ACh_ (measured carbachol sensitive current from 0 to the current at end of voltage step) by 66.5 ± 3.1% (n = 5, P < 0.01 vs control). However, no significant inhibition of hKv1.5, hKv4.3, or I_K.ACh_ was observed with 30 μM acacetin prodrug (n = 5 for each group, P = NS). One the other hand, our previous study showed that 30 μM acacetin had ~50% inhibition of hERG current^10^, while 30 μM acacetin prodrug had no such effect on hERG channels expressed in HEK 293 cells (n = 4, data not shown). The results indicate that acacetin prodrug cannot inhibit the cardiac ion channels directly like the parent drug acacetin. The results from this study are consistent with previous reports for many other prodrugs^15^,^18^,^19^, which show that the prodrug is inactive *ex vivo* and needs to be converted into the parent active compound by a specific enzyme. The bioconversion of acacetin prodrug *ex vivo* and *in vivo* was verified as below.

### Bioconversion of acacetin prodrug

**Figure 4:**
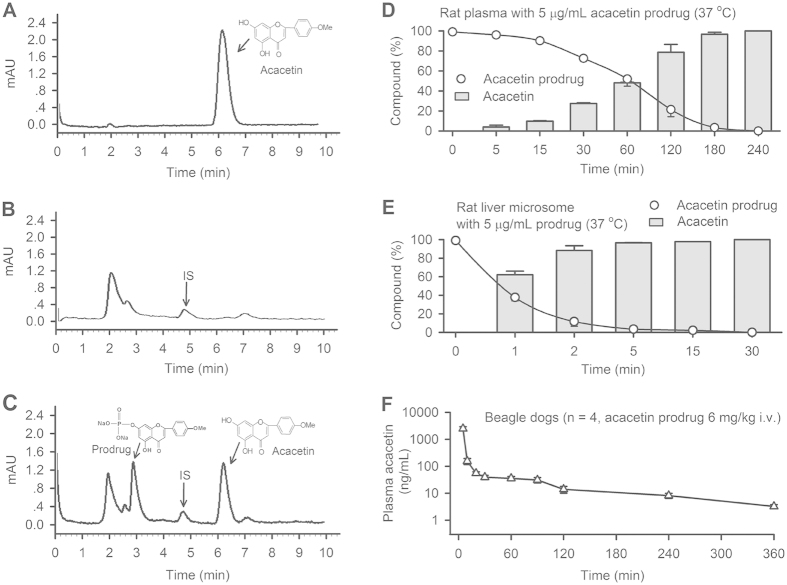
Bioconversion of acacetin prodrug. (**A**) HPLC graph of acacetin (1 μg/mL) in mobile phase. (**B**) HPLC graph of blank rat plasma with 50 ng/mL pentamethylquercetin as an internal standard (IS). (**C**) HPLC graph of rat plasma containing acacetin prodrug (1 μg/mL) and an IS (50 ng/mL) with 60 min incubation (37 °C). (**D**) Mean percentage values of acacetin prodrug and acacetin converted from acacetin prodrug in rat plasma (at different time points, n = 3). (**E**) Mean percentage values of acacetin prodrug and acacetin converted from acacetin prodrug in rat liver microsome (at different time points, n = 3). (**F**) Mean values of acacetin concentrations in plasma of beagle dogs (n = 4) administered intravenously with 6 mg/kg acacetin prodrug.

It is well recognized that phosphate ester-based prodrugs could be converted into parent compounds by alkaline phosphatase^15^,^19^ which is present in all tissues in the body^20^. The bioconversion of acacetin prodrug (Fig. 4) was determined initially in *ex vivo* rat plasma and rat liver microsome, and then in *in vivo* beagle dogs. Figure 4A shows a peak observed at 6.2 min on HPLC graph in mobile phase containing 1 μg/mL acacetin. No peak was observed at 6.2 min on HPLC graph in blank rat plasma, only an internal standard (IS) peak (pentamethylquercetin, 50 ng/mL) was observed at 4.7 min (Fig. 4B). A significant peak of acacetin was observed at 6.2 min in rat plasma with incubation of 1 μg/mL acacetin prodrug for 60 min (Fig. 4C), suggesting that acacetin prodrug is effectively converted into acacetin by alkaline phosphatase in rat plasma. In addition, the peaks at 2.9 and 4.7 min were also observed on HPLC graph for acacetin prodrug and IS, respectively (Fig. 4C).

Figure 4D illustrates the mean values of time-dependent conversion rate of acacetin prodrug in rat plasma (37 °C), in which 50% prodrug was converted into acacetin with 60 min incubation, reaching 100% conversion within 240 min incubation. Interestingly, the conversion speed of acacetin prodrug was greatly increased in rat microsome containing high level of alkaline phosphatase^20^, in which 60% conversion was observed with 1 min incubation, and 100% conversion was observed with 30 min incubation (Fig. 4E). These results suggest that acacetin prodrug can be effectively converted into acacetin in *ex vivo* rat plasma and rat liver microsome.

Figure 4F shows the mean values of plasma acacetin concentration at 5, 15, 30, 45, 60, 90, 120, 240 and 360 min after intravenous administration of acacetin prodrug (6 mg/kg, n = 4) in beagle dogs. Acacetin concentration was 2420 ng/mL at 5 min after the acacetin prodrug administration, and declined quickly thereafter. This result indicates that acacetin prodrug, as expected, could be converted into acacetin *in vivo* in beagle dogs.

### Pre-pharmacokinetic profile of acacetin

The pre-pharmacokinetic profile of acacetin was analyzed with the program (WinNonlin®1.3, Pharsight Co., Mountain View, CA, USA) in beagle dogs with an intravenous bolus of acacetin prodrug (6 mg/kg, Fig. 4F). The standard non-compartmental and compartmental pharmacokinetic parameters of acacetin such as C_max_ (maximum concentration), T_max_ (time to reach C_max_), T_1/2_ (time required for the concentration of the drug to reach half of its original value), CL (systemic clearance), AUC_∞_ (area under the respective first moment–time curve from time zero to infinity), AUC_last_, and other pharmacokinetic parameters of acacetin are summarized in Tables 1 and 2.

The concentration-time curve of acacetin was best fitted to a two-compartmental model, which could be expressed as: concentration (acacetin) = Ae^−α∙T^ + Be^−β∙T^, in which the A is zero-time intercept of the distribution slope in the compartment model; the B is zero-time intercept of decline in plasma concentration of drug; the α is distribution rate constant; the β is elimination constant. The CL obtained from non-compartmental and compartmental pharmacokinetic analysis is comparable, as is 325 and 287 mL/min/kg, respectively. These results indicate that the metabolization and/or elimination of acacetin are rapid in beagle dogs, and continuous infusion is required for anti-AF experiments.

In anti-AF experiment one third dose bolus of acacetin prodrug was applied in 2 min to obtain the immediate therapeutical concentration followed by continuous infusion of two third dose to maintain the potential effective therapeutic concentration. Three doses of acacetin prodrug (3, 6 and 12 mg/kg) were employed for anti-AF experiments in beagle dogs.

### Anti-AF of acacetin prodrug in beagle dogs

**Figure 5:**
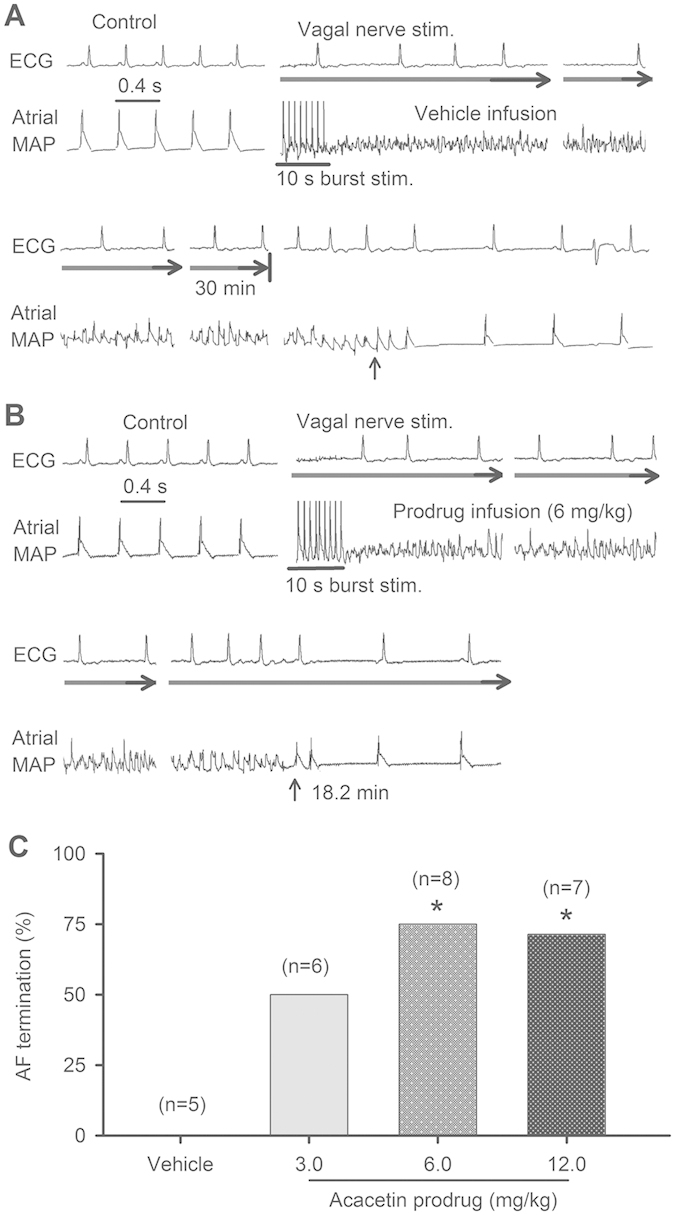
Anti-AF effect of acacetin prodrug. (**A**) Experimental AF induced by atrial burst pacing with vagal nerve stimulation (stim.) was not terminated (vertical arrow) by intravenous infusion of vehicle (5% glucose, 20 min) until withdrawing vagal nerve stimulation (thick horizontal arrows). (**B**) AF induced by atrial burst pacing and vagal nerve stimulation was terminated by intravenous infusion of acacetin prodrug (6 mg/kg, a bolus of one third dose in 2 min followed by continuous infusion of two third dose in 18 min). AF terminated at 18.2 min (vertical arrow) with continuous vagal nerve stimulation (thick horizontal arrows). (**C**) Percentage of AF termination by applying different doses of acacetin prodrug (*P < 0.05 vs. 5% glucose, χ^2^ test).

The anti-AF effect of acacetin prodrug was investigated in AF model (with AF duration of 30 min), which was established in beagle dogs with modified procedure as described previously^10^,^14^,^21^. After three-repeats of stable AF (30 min), drug treatment was applied on 4^th^ repeat (Fig. 5A). Acacetin prodrug dissolved in 5% glucose was administrated intravenously after 1 min of onset of AF induction. A bolus of one third dosage was infused within 2 min, then two third of the dosage (3, 6, and 12 mg/kg) was continuously infused for 18 min. The AF disappeared within 30 min before stopping bilateral vagal nerve stimulation was considered to be effective termination of AF (Fig. 5B). Blood samples were collected at different time points (5, 10, 20, 30, 45 and 60 min) during the experiment to determine the plasma acacetin concentrations. Figure 5C illustrates the termination rate of AF by different doses of acacetin prodrug. The termination rate of AF was 0% in animals treated with vehicle (5% glucose, n = 5) compared to 50% (3 of 6), 75% (6 of 8, P < 0.05 *vs*. vehicle) and 71.4% (5 of 7, P < 0.05 *vs*. vehicle) in animals treated with 3, 6 and 12 mg/kg acacetin prodrug, respectively. The AF duration in animals with effective AF termination was not significantly different in animals with different dosages of acacetin prodrug (19. 1 ± 3.1 min for 3 mg/kg, 17.2 ± 2.3 min for 6 mg/kg, and 15.5 ± 2.7 min for 12 mg/kg, P = NS). In addition, AF induction was repeated at 40 min after discontinuous prodrug infusion. AF was terminated at 25.3 ±1.8 min after re-induction (P < 0.01 vs. vehicle) in these animals (n = 15) with drug treatment, but not in animals treated with 5% glucose vehicle (30 min duration). However, the duration of AF was longer than during the initial drug administration (25.3 ± 1.8 min vs.17.1 ± 1.3 min, P < 0.01). These results indicate that intravenous infusion of acacetin prodrug can effectively terminate the AF in beagle dogs induced by vagal nerve stimulation and burst atrial pacing.

QTc (corrected QT) interval of ECG was analyzed in anesthetized beagle dogs before and at 35 min after drug administration. QTc interval was not altered (P = NS, before vs. after drug administration) in vehicle animals (n = 5, 273.6 ± 2.6 ms vs. 276.1 ± 4.2 ms) and animals treated with 3 mg/kg (n = 6, 279.5 ± 2.5 ms vs. 282.3 ± 1.7 ms), 6 mg/kg (n = 8, 276.9 ± 2.4 ms vs. 279.1 ± 2.3 ms), or 12 mg/kg (n = 7, 274.1 ± 3.5 ms vs. 278.7 ± 2.9 ms) acacetin prodrug. The results indicate that acacetin prodrug has no effect on QT interval.

**Figure 6:**
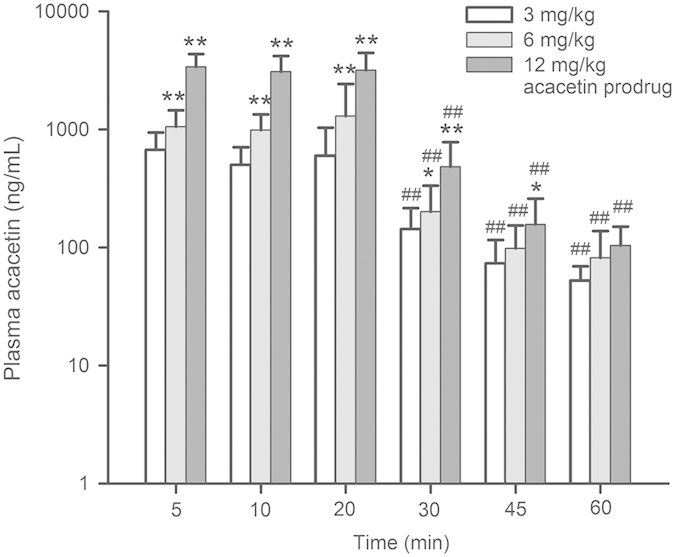
Plasma concentration profile of acacetin in beagle dogs treated with acacetin prodrug. Acacetin prodrug at 3 mg/kg (n = 6), 6 mg/kg (n = 8) and 12 mg/kg (n = 7) was intravenously administered with 1/3 of the dose as a bolus at 2 min, and 2/3 of the dose as a continuous infusion at 18 min. Plasma concentration declined rapidly after completing acacetin prodrug infusion (*P < 0.05 or P < 0.01 vs. 3 mg/kg; ^##^P < 0.01 vs. 5 min).

The plasma concentration profile of acacetin was determined with LC/MS/MS (liquid chromatography-tandem mass spectrometry) in animals with AF treatment. Figure 6 illustrates the mean plasma concentrations of acacetin converted from acacetin prodrug in beagle dogs treated with 3 mg/kg (n = 6), 6 mg/kg (n = 8), and 12 mg/kg (n = 7) acacetin prodrug, respectively. The plasma concentrations of acacetin declined rapidly after stopping infusion of acacetin prodrug, which further indicates that continuous infusion is required clinically to maintain a stable plasma concentration.

### Acute toxicity of acacetin prodrug

The experimental record is illustrated in Fig. S5A for the acute toxicity assessment in mice administered a single dose of acacetin prodrug through the tail vein. All animals survived within 24 h after receiving a dose of up to 540 mg/kg of acacetin prodrug. Conversely, all animals died after receiving a dose of 900 mg/kg of acacetin prodrug within 15~30 min. The dead animals generally presented an initial sedation, then suddenly excited spasm followed by death. No significant adverse effects or signs of toxicity were observed in the surviving animals during the observation. A necropsy of the surviving mice revealed no gross pathological alteration. The median lethal dose (or 50% lethal dose, LD_50_) calculated using Bliss method^22^,^23^ was 721.7 mg/kg in mice (Fig. S5B). The calculated LD_50_ with 95% confidence level was 685.1 to 760.2 mg/kg. By translating the dose based on body surface area^24^,^25^, the LD_50_ would be 108.3 mg/kg in dogs, which is much greater than the therapeutic dose of 3~12 mg/kg in beagle dogs. These results indicate that acacetin prodrug is a highly promising safe drug candidate with a wide therapeutic window to treat AF.

## Discussion

Prodrug is a well-established versatile strategy which provides possibilities for bioactive compounds to overcome various barriers to drug formulation and delivery, e.g. poor aqueous solubility, chemical instability, insufficient oral absorption, rapid pre-systemic metabolism, inadequate brain penetration, toxicity and local irritation, and also improves drug targeting of existing drugs with improved properties^15^,^16^. The inactive prodrug could be efficiently converted into the active form via *in vivo* chemical or enzymatic transformation. Thousands of patents were granted for the development of prodrugs, and more and more prodrugs have been approved for clinical application in treating different disorders^16^. The present study of acacetin prodrug represents the first druggable flavone among natural flavonoid compounds with wide *ex vivo* beneficial effects^26^, which can be intravenously administrated to treat AF in beagle dogs. This is likely an important step in potentially developing other water-soluble prodrugs of flavonoid compounds to treat patients with different disorders by injection administration.

There are several considerations in the design of “developable” prodrugs, including the kinds of functional groups involved, the complete bio-conversion rate *in vivo*, the stability of the prodrug and importantly the original pharmacological effect. The parent drugs could be modified by a polar, most often an ionizable promoiety, to increase water solubility. Phosphate esters-based prodrugs show good chemical stability^27^ while undergoing rapid and often quantitative cleavage *in vivo* via alkaline phosphatases and are widely used to synthesize prodrugs for improving the aqueous solubility, not only for oral drugs, but also for drugs intended for parenteral administration (e.g. intravenous administration)^15^,^18^,^19^. The phosphate group could either be linked to the parent drug directly where possible, or through a linker or “spacer” group. Although the use of “spacer” or “linker” group such as oxymethyl might decrease the steric hindrance around the enzymatically cleavable bond (phosphates, esters, carbonates) and consequently increase the bio-conversion rate^28^, the possible systemic release of the toxic compound formaldehyde is unwanted^15^,^29^,^30^. Fortunately, Acacetin (compound 1, Fig. 1) is a natural flavone with two active hydroxyl on the chromone backbone that can be modified on the 5-OH and 7-OH. In the present study, we managed to prepare a phosphate ester based acacetin prodrug with good water solubility by conjugated the phosphate group with the 7-OH of acacetin.

The aqueous solubility of acacetin, as other flavone compounds^26^, is very low (at nanomolar levels). Acacetin prodrug (purity >99%) is highly soluble in H_2_O and clinically acceptable solutions of 5% glucose and 0.9% NaCl. Because acacetin is a unique flavone with selective blockade of cardiac K^+^ currents^10^,^11^,^12^,^14^, effects of acacetin prodrug on these currents were therefore verified in *ex vivo* cell models. As many other prodrugs^15^,^18^,^19^, acacetin prodrug is inactive in *ex vivo* pharmacological tests. It (30 μM) had no blocking effect on hKv1.5, hKv4.3, or I_KACh_, whereas acacetin at 10 μM remarkably inhibited these ionic currents. The bioconversion was therefore required for the prodrug into the parent compound. Indeed, we observed that acacetin prodrug was gradually converted into acacetin in *ex vivo* incubation (37 °C) of rat plasma. The quick conversion was also observed in rat microsome incubation or in whole-animal (beagle dog) administration.

With intravenous administration of acacetin prodrug, the pre-pharmacokinetic profile of acacetin analysed with the standard non-compartmental and compartmental parameters showed that the metabolization and/or elimination of acacetin are rapid in beagle dogs. We therefore infused continuously acacetin prodrug in anti-AF experiment, in which the dose regimen was made with one third dose bolus within 2 min to obtain the immediate therapeutical concentration followed by continuous infusion of two third doses within 18 min to maintain the effective therapeutic concentration of acacetin to inhibit atrial I_Kur_ and I_KACh_, as well as cardiac I_to_. Intravenous infusion of acacetin prodrug (3~12 mg/kg) terminated AF induced by atrial bursting pacing and vagal nerve stimulation with efficacy of 50~75% and the effect can still be observed 40 min after discontinued drug infusion. This suggests that acacetin prodrug may be a promising drug candidate that can be used in clinic to treat patients with acute AF.

It is interesting to note that acacetin prodrug had no effect on QTc interval. On the other hand, the acute toxicity assessment in mice demonstrated a high LD_50_ of 721 mg/kg with intravenous administration. With the dosage translation based on the body surface area^24^, the LD_50_ in dogs would be 108 mg/kg (i.v.), which is far less than the therapeutic doses (3~12 mg/kg), suggesting that acacetin prodrug is a safe drug candidate.

One of limitations was that the acacetin prodrug underwent a rapid metabolism after a bolus injection. The intravenous infusion application may maintain consistent therapeutic plasma levels; however, more effort is required pharmaceutically to slow the metabolic release from the prodrug. In addition, though rapid atrial burst pacing-induced AF model with vagal nerve stimulation is widely used to test the efficacy of anti-AF drugs, it should be noted that this model may not represent general mechanisms of AF in humans. More effort is required for a next step to test efficacy of the acacetin prodrug against AF induced by other AF models and to obtain more comparable data for a clinical scenario. Nonetheless, these would not affect the conclusion that the synthesized water-soluble prodrug of acacetin is effective in terminating AF induced by vagal nerve stimulation.

## Conclusion

The present preclinical study demonstrated for the first that the synthesized highly water-soluble acacetin prodrug could be converted into the parent compound acacetin and exert an effective action in terminating the experimental AF induced by atrial burst pacing with vagal nerve stimulation in beagle dogs. Acacetin prodrug may be a promising drug candidate for clinic trial in treating patients with acute AF.

## Materials and Methods

### Preparation of phosphate sodium prodrug of acacetin

The phosphate sodium salt prodrug of acacetin (“acacetin prodrug” in short) was synthesized with acacetin (Fig. 1, compound 1) and dibenzylphosphite through a series of chemical reactions. A yellow solid of the intermediate compound (Fig. 1, compound 2) was initially synthesized after the reaction (7-OH) of acacetin with dibenzylphosphite in dimethylformamide (DMF) and CCl_4_, followed by mixture evaporation, and column chromatography on silica gel using n-hex/EtOAc/CH_2_Cl_2_. Phosphate ester of acacetin (Fig. 1, compound 3) was then produced by stirring reaction of compound 2 with formic acid and Pd/C in MeOH and tetrahydrofuran (THF) for 2 h. After filtering and concentrating the reaction mixture, compound 3 was obtained as yellow solid. Acacetin prodrug (Fig. 1, compound 4) was finally produced by vigorously stirring compound 3 in H_2_O while slowly adding NaHCO_3_. The mixture was stirred at 30 °C until most of the compound dissolved. A light yellow solid of acacetin prodrug was precipitated by washing and filtering with ethanol and drying. The detailed synthesis procedure is described in Supplemental Materials, and the products were characterized and confirmed by ^1^H Nuclear Magnetic Resonance (^1^H NMR, Figs S1–S3).

### Aqueous solubility of acacetin prodrug in different solutions

The aqueous solubility of acacetin, the intermediate phosphate ester of acacetin, or acacetin prodrug was determined at room temperature (23–25 °C) in dH_2_O clinically acceptable vehicles 5% glucose or saline (0.9% NaCl) with high performance liquid chromatography (HPLC) as described in Supplemental Materials.

### Effects of acacetin prodrug on cardiac potassium channels

Whole-cell currents of hKv1.5 and hKv4.3 were recorded in established HEK 293 cell lines stably expressing hKv1.5 (KCN5A) and hKv4.3 (KCND3) and I_KACh_ was determined in isolated rat atrial myocytes as described previously^12^,^31^,^32^ and in Supplemental Materials.

### Bioconversion of acacetin prodrug

The *ex vivo* bioconversion of acacetin prodrug was determined in rat plasma or rat hepatic microsome that contains high content alkaline phosphatase^33^, as described previously^30^ and in Supplemental Materials. The *in vivo* bioconversion of acacetin prodrug was determined in beagle dogs as described in Supplemental Materials. The protocols of animal experiments were approved by the Ethics Committee of Animal Care and Use for Teaching and Research of the University of Hong Kong, and all experiments were performed in accordance with the approved guidelines

### Pre-pharmacokinetics of acacetin in beagle dogs

The pre-pharmacokinetic parameters of acacetin were calculated in beagle dogs with intravenous administration of 6 mg/kg acacetin prodrug using non-compartmental analysis and compartmental analysis using WinNonlin®1.3 (Pharsight Co., Mountain View, CA, USA) as described in Supplemental Materials.

### Vagal nerve stimulation-induced AF in beagle dogs

Experimental AF was induced by vagal nerve stimulation in beagle dogs with modified procedure as described previously^10^,^14^,^21^. Briefly, AF was generated by a 10-s burst atrial pacing with a 4-fold diastolic threshold potential (60-ms interval) after 30 s vagal nerve stimulation. If AF persisted for 30 min, it was terminated by stopping vagal nerve stimulation. The stable AF lasting for 30 min was repeated for three times (20 min interval), drug test (vehicle or different doses of acacetin prodrug) was then performed. A shortened duration of AF during the continuous vagal nerve stimulation was considered to be effective termination of AF for drug treatment.

### Acute toxicity assessment of acacetin prodrug

The acute toxicity of acacetin prodrug was assessed in ICR mice (weighing 18~22 g) as described in Supplemental Materials, and the LD_50_ was calculated by the Bliss method^22^,^23^.

### Data analysis

All results are expressed as mean ± SEM. Statistical comparisons were analyzed by paired Student *t* test or repeated ANOVA where appropriate. Categorical data were analyzed with the χ^2^ test. A value of *P < *0.05 was considered statistically significant.

## Additional Information

**How to cite this article**: Liu, H. *et al*. Synthesis of a highly water-soluble acacetin prodrug for treating experimental atrial fibrillation in beagle dogs. *Sci. Rep*. **6**, 25743; doi: 10.1038/srep25743 (2016).

## Supplementary Material

Supplementary Information

## Figures and Tables

**Table 1:** Non-compartmental pharmacokinetic parameters of acacetin converted from acacetin prodrug (6 mg/kg, i.v.) in beagle dogs (n = 4, mean ± SEM).

Pharmacokinetic parameters	Acacetin
**T_max_** (min)	5
**C_max_** (ng/ml)	2483 ± 420
**T_1/2_** (min)	95.0 ± 7.78
**AUC_last_** (min*ng/ml)	19351 ± 3407
**AUC_∞_** (min*ng/ml)	19360 ± 3345
**MRT_last_** (min)	34.6 ± 2.51
**CL** (ml/min/kg)	325 ± 49.9
**V_ss_** (ml/kg)	45069 ± 9046
**AUMC_∞_** (min*min*ng/ml)	687086 ± 163867

AUC_last_, area under the plasma concentration-time curve; AUC_∞_, area under the plasma concentration-time curve from time zero to infinity; AUMC_∞_, area under the first moment of plasma concentration-time curve from time zero to infinity; C_max_, maximum (peak) concentration of a drug after administration; T_max_, Time to reach C_max_; MRT, mean residence time; T_1/2_, time required for the concentration of the drug to reach half of its original value; CL, systemic clearance; V_SS_, apparent volume of distribution at steady-state.

**Table 2:** Compartmental pharmacokinetic parameters of acacetin converted from acacetin prodrug (6 mg/kg, i.v.) in beagle dogs (n = 4, mean ± SEM).

Pharmacokinetic parameters	Acacetin
*K10_HL* (min)	12.8 ± 5.8
*Alpha* (1/min)	0.0800 ± 0.0735
*Beta* (1/min)	0.0
A (ng/mL)	2382 ± 3170
B (ng/mL)	5.10 ± 9.12
CL (mL/min/kg)	287 ± 103
V_ss_ (mL/kg)	12526 ± 6338
V_2_ (mL/kg)	6609 ± 3230

*K10_HL*, a constant of elimination rate; *Alpha*, an initial phase of rapid decrease in plasma concentration; *Beta*, a phase of gradual decrease in plasma concentration after the alpha phase; CL, systemic clearance; V_SS_, apparent volume of distribution at steady-state; V_2_, apparent volume of distribution of peripheral compartment.
