# Supplementary material for: Synthesis of a highly water-soluble acacetin prodrug for treating experimental atrial fibrillation in beagle dogs
Source: Sci Rep. 2016 May 10;6:25743. doi: 10.1038/srep25743 (PMC4861903; doi:10.1038/srep25743)
Supplement: Supplementary Information [file srep25743-s1.pdf]

Supplementary materials

Synthesis of a highly water-soluble acacetin prodrug for treating experimental atrial fibrillation in beagle dogs

Hui Liu, Ya-Jing Wang, Lei Yang, Mei Zhou, Man-Wen Jin, Guo-Sheng Xiao, Yan Wang, Hai-Ying Sun, Gui-Rong Li

Supplementary methods

Synthesis of phosphate sodium salt prodrug of acacetin.

To a solution of acacetin (Fig. 1, Compound 1, 3 g, 10.5 mM) and 4.5 mL of NEt_3 (32 mM) in DMF (20 mL) at 0 °C, 3.05 g of dibenzyl phosphite (11.6 mM) in CCl_4 (5.5 mL) was added dropwise and the mixture was stirred for 2 h at room temperature (22-23 °C). The mixture was evaporated and the crude product was purified by column chromatography over silica gel by using n-hex/EtOAc/ CH_2Cl_2 (6:1:2 v/v). After evaporating the solvent, 1.3 g of yellow solid of compound 2 (Fig. 1) was obtained.

To a solution of compound 2 (2.1 g, 3.9 mM) and formic acid (0.1 mL) in MeOH (20 mL), THF (10 mL) was added with 10% Pd/C (0.2 g) at 1 atm. After stirring for 2 h, Pd/C was filtered. The filtrate was concentrated and 1.3 g of yellow solid of compound 3 (Fig. 1, phosphate ester of acacetin) was obtained.

To Compound 3 (5.0 g, 549.09 mM, 1.00 Eq) in H_2O (100 mL), $NaHCO_3$ (1 M, 1.15 L, 2.10 Eq) was added drop-wise at 5 ℃, and then the mixture was heated to 30 ℃ for 4h, till most of bubbles disappeared and the solution was clear. The solution was cooled to 25 ℃, and extracted with EtOAc (200 mL * 4) until HPLC showed the purity of the aqueous phase was more than 99%. The aqueous phase was concentrated to 50 mL, freeze-dried to give compound 4 (acacetin prodrug, 5.1 g, 92% yield, 99.1% purity) as a light yellow solid.

Aqueous solubility of the acacetin prodrug in different solutions

Water solubility of acacetin, the intermediate phosphate ester of acacetin and the acacetin prodrug were determined at room temperature in dH_2O, clinically acceptable vehicles 5% glucose and saline (0.9% NaCl). A fixed amount (20 mg) of the compounds was added to different vehicles, and mixed with a vortex for 2 min. The mixture was then sonicated for 60 min and centrifuged at 12000 *g* for 30 min. The supernatant was collected and diluted with methanol (for acacetin, 1:1) or other solvents accordingly (phosphate ester of acacetin and acacetin prodrug, 1:10000). The diluted mixture was filtered (0.22 μm Millipore), and analyzed by HPLC (for acacetin) or UV spectrometer (for phosphate ester of acacetin and acacetin prodrug). All experiments were performed in triplicate.

The HPLC system was equipped with a C-18 column (Grace, 4.6x150mm, 5 μm) and the

mobile phase was composed of a mixture of water and methanol (v/v, 3:7, with 0.2% phosphate acid) using an isocratic elution (Hitachi L2130 pump, L2200 Autosampler and L2400 Detector, Hitachi, Japan). The flow rate was 1.0 mL/min. The UV absorbance detector was set at 330 nm. Pentamethylquercetin (50 ng/mL) was employed as an internal standard (IS). The sample injection volume was 20 μL. The retention time of acacetin prodrug, pentamethylquercetin, and acacetin were 2.9, 4.7 and 6.2 min, respectively.

The standard curves for acacetin, phosphate ester of acacetin and acacetin prodrug were linear over the concentration range of 50-5000 ng/mL (γ^2=0.9997), 50-2000 ng/mL (γ^2=0.9952) and 1-100 μg/mL (γ^2=0.9997), respectively. The representative regression equation were $y = 381.45x + 5000.3$, $y = 174.24x – 7841$ and $y = 0.0031x + 0.0027$, respectively.

Effects of acacetin prodrug on cardiac potassium currents

Whole-cell currents of hKv1.5 and hKv4.3 were recorded in established HEK 293 cell lines stably expressing hKv1.5 (KCN5A) and hKv4.3 (KCND3).[1, 2] The cells were cultured in Dulbecco's modified eagle's medium (DMEM, Invitrogen, Hong Kong) supplemented with 10% fetal bovine serum and 400 μg/mL G418. Cells on a coverslip were transferred to an open cell chamber (0.5 ml) mounted on the stage of an inverted microscope and superfused with Tyrode's solution at 2 mL/min. The whole cell patch-clamp technique was used as described previously.[1, 2] The whole-cell membrane currents were measured using an EPC-10 amplifier and Pulse software (Heka Elektronik, Lambrecht, Germany). Borosilicate glass electrodes (1.2-mm OD) were pulled with a Brown/Flaming puller (model P-97, Sutter Instrument, Nato, CA) and had resistances of 1.5~2.5 MΩ when filled with the pipette solution. A 3-M KCl agar bridge was used as the reference electrode. The tip potential was zeroed before the patch pipette contacted the cell. After a giga-Ohm seal was obtained, the cell membrane was ruptured by applying gentle pressure to establish a whole-cell configuration. Series resistance (Rs) was 3~5 MΩ and was compensated by 50–70% to minimize voltage errors. The liquid junction potential (14.7 mV) calculated with the software Clampex was not corrected in the experiment and data analysis.

Whole-cell current of I_{KACh} was determined in isolated rat atrial myocytes. The myocytes were dissociated from male Sprague-Dawley rats (230-300 g, n = 5), were obtained from Laboratory Animal Unit of University of Hong Kong. The protocol was approved by Ethic Committee of Animal Use for Teaching and Research of University of Hong Kong. Atrial myocytes from rat hearts were enzymatically dissociated by the procedure described previously.[3] The isolated myocytes were kept in a high potassium medium at room temperature for 1 h before electrophysiological recording.[4] After whole-cell membrane current reached stable, 5 μM carbachol was added to elicit I_{KACh} in rat atrial myocytes. Current and voltage signals were low-pass filtered at 5 kHz and stored in the hard disk of an IBM compatible computer. All experiments were conducted at room temperature.

Bioconversion of acacetin prodrug in rat plasma and hepatic microsomes

Hydrolysis of acacetin prodrug was initially determined in rat plasma. The 100 μL rat plasma samples containing 5 μg/mL prodrug was prepared at 4 °C and then incubated in a water bath at 37 °C and continuously shaken at 60 rpm. The plasma samples were collected at 0, 5, 15, 30, 60, 120, 180, 240 and 360 min, respectively. The reaction was terminated by adding 1 mL cold methanol containing the IS solution (50 ng/mL pentamethylquercetin). The plasma treatment was described below.

Bioconversion of acacetin prodrug was also determined in rat hepatic microsomes containing high content alkaline phosphatase,[5] as described previously.[6] Sprague-Dawley rat liver microsomes were purchased from Invitrogen (Hong Kong, China). The reaction mixture was prepared in 50 mM Tris-HCl buffer (pH = 7.4) with 1 mM NADPH (nicotinamide adenine dinucleotide phosphate) and 0.5 mg/mL (total protein) microsomes. Prodrug solutions were added to the microsomes reaction mixture with a final concentration of 5 μg/mL. The reaction solution was incubated in a water bath at 37℃ and shaken at 60 rpm for 1 h. The samples were collected at 0, 1, 2, 15, 30 and 60 min. The reaction was terminated by adding 200 μL cold methanol containing internal standard (pentamethylquercetin, 50 ng/ml). After centrifugation at 12000 g for 30 min, the supernatant was immediately analyzed by HPLC. The microsome standards of prodrug and acacetin were prepared by mixing of different amount of acacetin prodrug and acacetin with 100 μL of blank rat microsome to give final prodrug and acacetin concentrations of 500-5000 ng/mL respectively. The standard curves for acacetin and prodrug in rat microsome were made as described above. The representative regression equation were y = 401.54x + 14326 for acacetin, y = 110.08x - 8842.7 for prodrug over the concentration range of 500-5000 ng/mL (γ^2=0.997).

The extent of the prodrug conversion into acacetin was expressed by equation-conversion percentage (%) = $\frac{C_{Pro-1}^{0}-C_{Pro-1}}{C_{Pro-1}^{0}}$ x 100, where the C_{Pro-1}^{0} was the concentration (by HPLC) of prodrug in the plasma at 0 min, the C_{Pro-1} was the concentration of prodrug at time points.

Bioconversion of acacetin prodrug in beagle dogs

The protocol of animal experiments was approved by the Ethics Committee of Animal Care and Use for Teaching and Research of the University of Hong Kong. Acacetin prodrug (10 mg/mL in 5% glucose) was intravenously administered in beagle dogs (6 mg/kg) in 2 min. Blood samples (~0.5 mL) were collected from the fore or hind limbs vein before and after 5, 15, 30, 60, 90, 120, 240 and 360 min of acacetin prodrug administration. The plasma was separated by centrifugation of whole blood (5,000 rpm, 20 min) and then was stored at −25°C until analysis. The plasma (10 μL) was mixed with 10 fold methanol (100 μL) and vortexed for 5 min to precipitate plasma protein, followed by centrifugation at 12,000 g for 30 min. The final supernatant (1 μL) was injected into UPLC/MS/MS system (Shimadzu, Tokyo, Japan) for

analysis. In addition, three quality control samples (QCs) at 1, 25 and 400 ng/mL were prepared in blank plasma.

The analytical method was validated according to the FDA guidelines for bioanalytical method validation (US Food and Drug Administration, 2001). Mass spectrometric data were acquired in positive electrospray ionization mode with the following source parameters: Ion Spray voltage, 5500 V; temperature, 550 ℃; curtain gas, 25; ion source gas 1, 45; and ion source gas 2, 45. Data were recorded in multiple reaction monitoring mode. The mass transitions chosen for the quantitation of the compounds were m/z 285.0→m/z 241.8 for acacetin and m/z 373.0→m/z 312.0 for the IS. The retention time of IS and acacetin was 1.97 and 2.02 min, respectively.

Linearity range with 1/x/x weighting was from 1~500 ng/mL. The lower limit of quantification was 0.5 ng/mL, and the limit of detection was 0.1 ng/mL for acacetin. Assay accuracy at low, medium, and high QCs was 100 ± 3.2%, 103.6 ± 2.9% and 97.5 ± 5.1% respectively (n = 5). Samples were stable within 4 h on the auto-sampler. The peak concentration (C_{max}) and time to reach C_{max} (T_{max}) were read directly from individual acacetin plasma concentration-time profiles. The mean values of plasma concentrations were calculated and plotted against the time points.

Pre-pharmacokinetics of acacetin in beagle dogs

The pharmacokinetic parameters were calculated using non-compartmental analysis and compartmental analysis respectively using WinNonlin®1.3 (Pharsight Co., Mountain View, CA, USA). Thus, the area under the plasma concentration-time curve (AUC_{last}) was calculated using the linear trapezoidal method from 0 to last sampling time. The area under the plasma concentration-time curve from zero to time infinity (AUC_{∞}) was calculated as $AUC_{last} + C_t/\lambda$, where C_t refers to the last measured concentration and λ represents the slope of the log-linear portion of the concentration time profile. The area under the respective first moment-time curve from time zero to infinity ($AUMC_{\infty}$) was calculated using the linear trapezoidal method and appropriate area extrapolation as described by Gibaldi and Perrier.[7] Standard methods were used to calculate the time-averaged total body clearance (CL) and the apparent volume of distribution at steady-state (V_{SS}) by a non-compartmental analysis. The systemic clearance (CL) and the volume of distribution at V_{SS} were estimated using the following equations:

$$\mathrm{CL} = \frac{Dose}{AUC_{\infty}} \qquad and \qquad \mathrm{V}_{ss} = MRT_{\infty} \times \mathrm{CL}$$

MRT (Mean residence time) was calculated with $MRT = \frac{AUMC}{AUC}$.

Vagal nerve stimulation-induced atrial fibrillation in Beagle dogs

Beagle dogs (either gender, 10~12 kg, Wuhan Anlu Experimental Animal Laboratory, Wuhan,

China) were anesthetized with pentobarbital (40 mg/kg, i.v.), supplemented as necessary during the experiment. The animal was kept on an operation table maintained at 37 ℃, intubated and ventilated with room air. The left femoral vein was cannulated to maintain fluid balance, anesthesia, and test articles. Lead II electrocardiogram (ECG) was continuously monitored with using a digital recording system (RM6240, Chengdu Instruments, China)

An action potential (MAP) recording- and pacing-catheter (Boston Scientific Ltd) was introduced to the right atrium through right jugular vein, and an electrical pacing catheter was introduced to left ventricle via left jugular artery for ventricular pacing (when necessary). Bilateral cervical vagal nerves were isolated and bipolar electrodes were placed to stimulate efferent vagal nerves with 15 Hz, 0.2 ms duration voltage step (80-100% threshold) to induce a 50% reduction of heart rate. Ventricular pacing was performed with 2-ms voltage pulses (2.5 Hz, 150% diastolic threshold) when vagal nerves were continuously stimulated. Experimental atrial fibrillation (AF) model is established in beagle dogs with modified procedure as described previously.[8-10] Briefly, AF was generated by a 10-s burst atrial pacing with a 4-fold diastolic threshold potential (60 ms interval) after 30 s vagal nerves stimulation. If AF persisted for 30 min, AF was terminated by stopping vagal nerve stimulation. After stable persistent AF lasing for 30 min was repeated for three times (20 min interval), drug test (vehicle or different doses of phosphate sodium salt prodrug of acacetin) was performed. A shortened AF duration during the continuous vagal stimulation was considered to be effective termination of AF. All the biological signals were recorded using the digital recording system (RM6240, Chengdu Instruments, China). QTc (corrected QT) intervals of electrocardiogram were determined in anesthetized beagle dogs before and at 35 min after drug administration using the Fridericia formula $QTc = QT/\sqrt[3]{RR}$, where RR is the mean of 10 R-R intervals

Acute toxicity assessment of acacetin prodrug

The acute toxicity of acacetin prodrug was assessed in ICR mice (weighing 18~22 g) provided by the Laboratory Animal Center of Hong Kong University (Hong Kong, China) and maintained under controlled temperature conditions (23 ℃), with a constant 12 h light-dark cycle and free access to food and water. Sixty ICR mice (male, 30; female, 30) were stratified by weight and randomly assigned to six groups: (n = 10 in each group, 5 male and 5 female). Acacetin prodrug dissolved in 5% glucose was intravenously administered at 900, 810, 765, 720, 630 and 540 mg/kg through tail vein, respectively. The animals were closely observed for symptoms and mortality in 24 h. All of the surviving animals were euthanized at the end of the study, and their vital organs were individually observed for gross pathology by necropsy, and the LD50 was calculated by the Bliss method.[11, 12]

References

1. Xu XH, Sun HY, Zhang YH, et al. Allitridi inhibits multiple cardiac potassium channels expressed in hek 293 cells. *PLoS One* **7,** e51550 (2012)

2. Wu HJ, Wu W, Sun HY, et al. Acacetin causes a frequency- and use-dependent blockade of hkv1.5 channels by binding to the s6 domain. *J Mol Cell Cardiol* **51,** 966-973 (2011)

3. Liu Y, Xu XH, Liu Z, et al. Effects of the natural flavone trimethylapigenin on cardiac potassium currents. *Biochem Pharmacol* **84,** 498-506 (2012)

4. Liu H, Sun HY, Lau CP, Li GR. Regulation of voltage-gated cardiac sodium current by epidermal growth factor receptor kinase in guinea pig ventricular myocytes. *J Mol Cell Cardiol* **42,** 760-768 (2007)

5. Kaplan MM, Righetti A. Induction of rat liver alkaline phosphatase: The mechanism of the serum elevation in bile duct obstruction. *J Clin Invest* **49,** 508-516 (1970)

6. Lang BC, Yang J, Wang Y, et al. An improved design of water-soluble propofol prodrugs characterized by rapid onset of action. *Anesth Analg* **118,** 745-754 (2014)

7. M. G, Perrier D. *Pharmacokinetics,*. New York: Marcel Dekker; 1982.

8. Machida T, Hashimoto N, Kuwahara I, et al. Effects of a highly selective acetylcholine-activated K^+ channel blocker on experimental atrial fibrillation. *Circ Arrhythm Electrophysiol* **4,** 94-102 (2011)

9. Li GR, Wang HB, Qin GW, et al. Acacetin, a natural flavone, selectively inhibits human atrial repolarization potassium currents and prevents atrial fibrillation in dogs. *Circulation* **117,** 2449-2457 (2008)

10. Li GR, Lau CP, Qin GW, H.B. W. Use of acacetin and related compounds as potassium channel inhibitors. *US Patent and Trademark Office (USPTO)* USA patent US Patent and Trademark Office (USPTO): 7,816,400 (2010).

11. Bliss CI. The method of probits. *Science* **79,** 38-39 (1934)

12. Raj J, Chandra M, Dogra TD, Pahuja M, Raina A. Determination of median lethal dose of combination of endosulfan and cypermethrin in wistar rat. *Toxicol Int* **20,** 1-5 (2013)

Supplemental figures

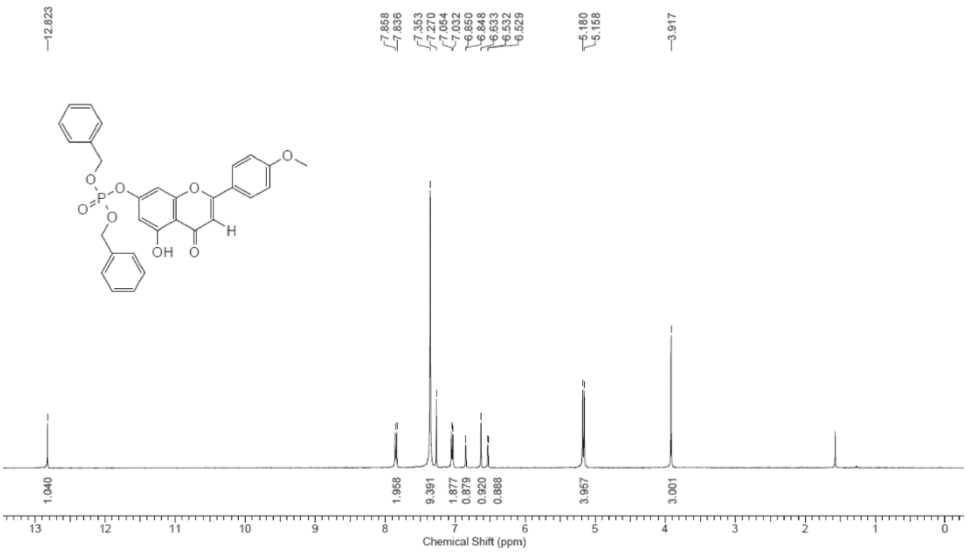

Figure S1 ^{1}H NMR spectra of compound 2.

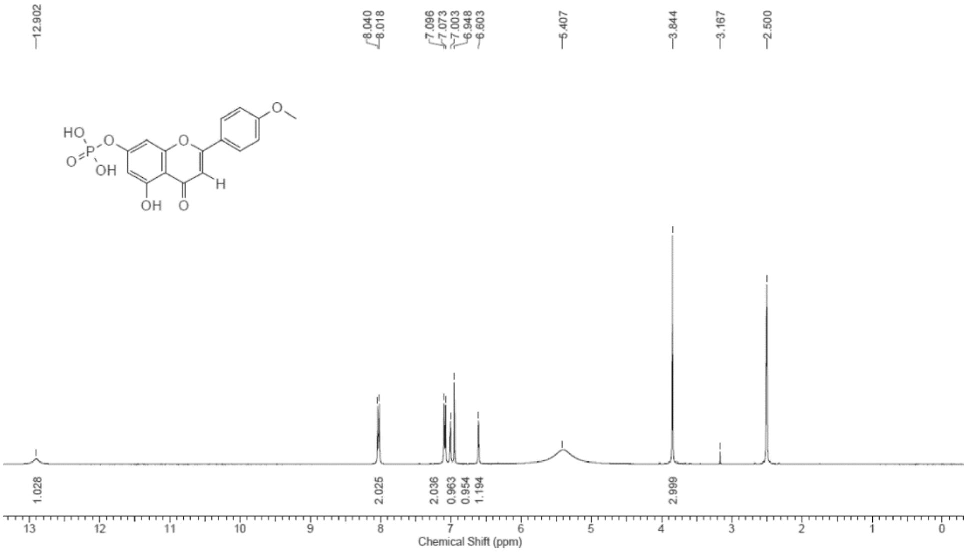

Figure S2 ^{1}H NMR spectra of compound 3 (phosphate ester of acacetin).

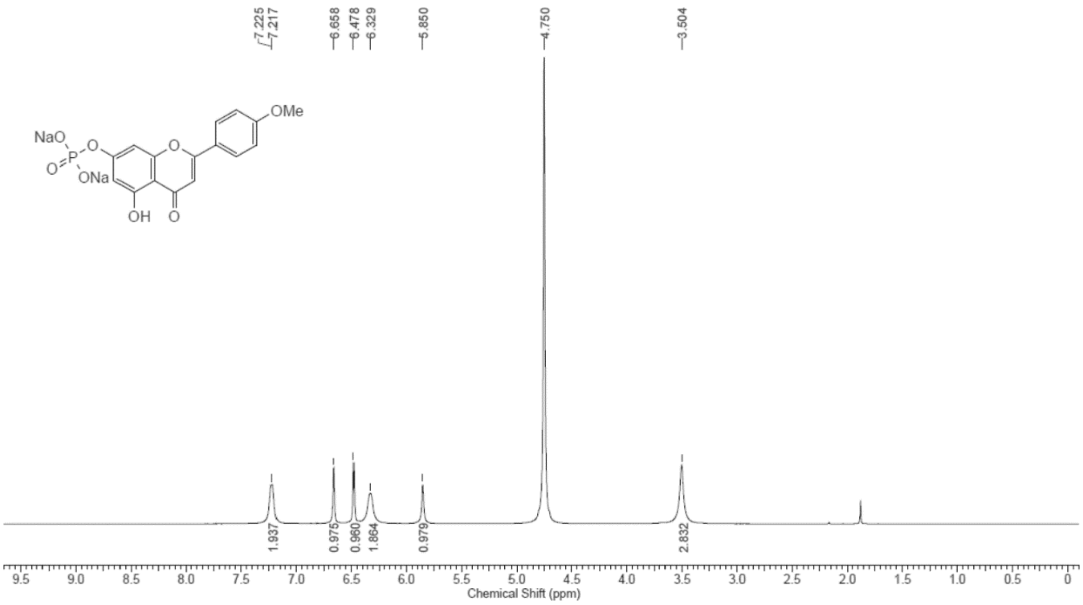

Figure S3. ^{1}H NMR spectra of acacetin prodrug.

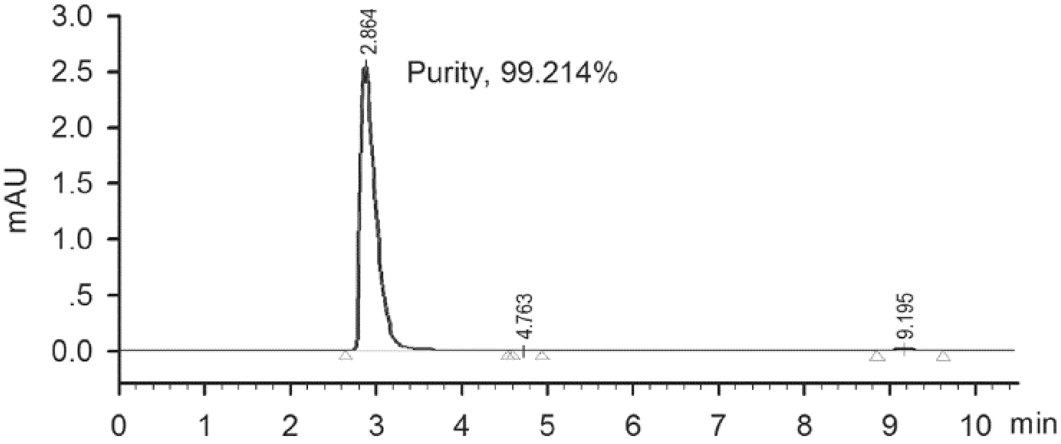

Figure S4. Purity of acacetin prodrug was determined with HPLC. Three peaks with integrated areas of 99.214%, 0.251% and 0.545%, respectively at retention times of 2.9, 4.8, and 9.2 min. Acacetin prodrug (at retention time of 2.9 min) has a high purity of >99%.

A Experimental record

Group	Animal (n)	Dosage (mg/kg)	Log dose	Death (n)	Mortality (%)
1	10	900	2.95	10	100
2	10	810	2.91	8	80
3	10	765	2.88	7	70
4	10	720	2.86	4	40
5	10	630	2.80	2	20
6	10	540	2.73	0	0

B

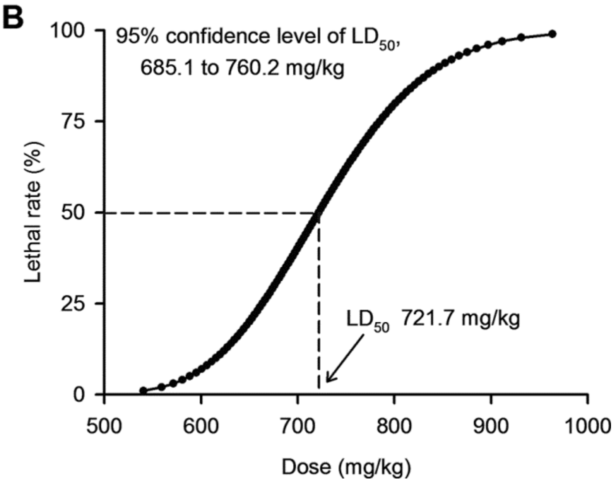

Figure S5. Acute toxicity of acacetin prodrug in mice. **A.** Experimental record for the result information. **B.** Curve fitting with Bliss method to obtain the LD_{50} value of 721.7 mg/kg in mice.
